# Geographically Diverse Clusters of Nontoxigenic *Corynebacterium diphtheriae* Infection, Germany, 2016–2017

**DOI:** 10.3201/eid2407.172026

**Published:** 2018-07

**Authors:** Alexandra Dangel, Anja Berger, Regina Konrad, Heribert Bischoff, Andreas Sing

**Affiliations:** Bavarian Health and Food Safety Authority, Oberschleissheim, Germany (A. Dangel, A. Berger, R. Konrad, H. Bischoff, A. Sing);; German Consiliary Laboratory on Diphtheria, Oberschleissheim (A. Berger, R. Konrad, A. Sing)

**Keywords:** bacterial infections, bacteria, diphtheria, drug users, homeless, disease outbreaks, molecular typing, epidemiology, DNA sequencing, public health, Germany

## Abstract

From 2016 through the middle of 2017, the German Consiliary Laboratory on Diphtheria noted an increase in nontoxigenic *Corynebacterium diphtheriae* isolates submitted from cities in northern Germany. Many patients for whom epidemiologic data were available were homeless, alcohol or drug abusers, or both. After performing routine diagnostics and multilocus sequence typing (MLST), we analyzed isolates of sequence type (ST) 8 and previously submitted isolates by whole-genome sequencing. Results were analyzed for phylogenetic relationship by core genome MLST (cg-MLST) and whole-genome single-nucleotide polymorphism profiles. Next-generation sequencing–based cg-MLST revealed several outbreak clusters caused by ST8; the geographic focus was in the metropolitan areas of Hamburg and Berlin. To achieve enhanced analytical depth, we used additional cg-MLST target genes and genome-wide single-nucleotide polymorphisms. We identified patient characteristics and detected transmission events, providing evidence that nontoxigenic *C. diphtheriae* infection is a potential public health threat in industrialized countries.

Diphtheria and its causative pathogen, *Corynebacterium diphtheriae*, have been drifting out of focus in Western countries because of effective vaccination programs. In 1994, the World Health Organization aimed to eliminate these infections by the year 2000, but epidemics such as that in the former Soviet Union and outbreaks in other countries show that the goal of elimination by 2000 was not reached (*1*) and nationwide vaccination programs are still essential (*2*). Despite high vaccination coverage in most Western countries, cases of wound and bloodstream infections caused by *C. diphtheriae* are rising because vaccination with toxoid prevents only diphtheria toxin–associated symptoms. Therefore, *C. diphtheriae* is again causing severe public health problems in many Western industrialized countries (*1**,**3*). Health conditions associated with certain socioeconomic factors that enhance the risk for infection have been identified, including heart disease (*4*), cirrhosis, dental caries (*5*), diabetes mellitus (*6*), and skin colonization (*6*). The most serious risk factors are intravenous drug or alcohol abuse along with their various resultant health complications, as well as homelessness (*7**,**8*).

From the middle of 2016 through the middle of 2017, the German Consiliary Laboratory on Diphtheria noted an increase in nontoxigenic *C. diphtheriae* isolates submitted from diagnostic microbiological laboratories or local public health authorities in several cities of northern Germany, especially Berlin and Hamburg. A smaller number of isolates came from surrounding areas and other bigger cities in northwestern Germany. Although epidemiologic data were not available for each patient, many patients colonized or infected with nontoxigenic *C. diphtheriae* were reported to be homeless, abusers of alcohol or intravenous drugs, or any combination of these factors. To determine if an outbreak with a common strain was ongoing among members of this risk group in northern Germany, we characterized the isolates in more detail by conducting molecular typing analyses to study phylogenetic relationships in an approach with stepwise increased resolution.

As a first step, we performed multilocus sequence typing (MLST), which has developed over the past 20 years (*9*) as a standard molecular typing assay with clear and reproducible nomenclature for >90 bacterial pathogenic species, including the 2 potentially toxigenic species *C. diphtheriae* (*10*) and *C. ulcerans* (*11*). However, with current next-generation sequencing (NGS) techniques that enable highly parallel sequencing of whole bacterial genomes in a few days and within a reasonable budget, investigations of outbreaks or clinical events can benefit from this enhanced discriminatory power. Therefore, we used NGS together with core genome MLST (cg-MLST) as a second step, applying high discriminatory depth with clear nomenclature. This technique has been successfully applied in outbreak investigations of a broad range of bacteria (*12**–**14*) and has recently been used for *Corynebacterium* (*15*). To confirm and refine the results in highest resolution, we used phylogeny based on genome-wide single-nucleotide polymorphisms (SNPs). 

## Material and Methods

### Bacteriology

We cultured samples on 5% sheep blood and serum tellurite agar plates (both Becton Dickinson, Heidelberg, Germany). Colonies suggestive of coryneform bacteria were subjected to MALDI-TOF (matrix-assisted laser desorption/ionization-time of flight) mass spectrometry (Microflex; Bruker Daltonics, Bremen, Germany) and API Coryne (bioMérieux, Marcy-L’Ètoile, France) according to published methods (*16*) for species and biovar determination. We analyzed presence of the toxin gene by using a quantitative PCR approach (*17*).

### Sequencing and Data Analyses

For DNA extraction, we used isolates from blood agar plates to inoculate liquid overnight cultures in brain–heart infusion broth (Thermo Scientific, Schwerte, Germany). Cells were harvested, resuspended in Tris buffer with 50 mg/mL lysozyme, and incubated 30 min at 37°C. We extracted genomic DNA by using a modified protocol of the Maxwell 16 LEV Blood DNA Kit on the Maxwell 16 instrument (Promega, Mannheim, Germany), starting with the addition of 150 μL incorporation buffer, 200 μL lysis buffer, 30 μL proteinase K, and 10 μL10 mg/mL RNase A, followed by incubation for 2 h at 65°C and then addition of 300 μL lysis buffer and transfer to the instrument. Genomic DNA was eluted in Tris buffer.

Routine MLST was performed by PCR of the 7 target regions *atpA*, *dnaE*, *dnaK*, *fusA*, *leuA*, *odhA*, and *rpoB* by using a previously described protocol (*10*) and Sanger sequencing at GATC Biotech (Konstanz, Germany). Sequences were analyzed with the SmartGene IDNS (SmartGene, Lausanne, Switzerland). Unknown alleles were submitted to the *C. diphtheriae* PubMLST website (https://pubmlst.org/cdiphtheriae/; *18*) for assignment of new alleles or sequence types (STs).

Whole-genome libraries for NGS were prepared with the Nextera XT kit (Illumina, San Diego, CA, USA), and sequencing was performed with 2 × 250-bp paired-end reads on the Illumina MiSeq. Quality control of NGS sequencing runs was accomplished by using Illumina SAV software (http://emea.support.illumina.com/sequencing/sequencing_software/sequencing_analysis_viewer_sav.html). 

We generated a *C. diphtheriae* cg-MLST scheme, defining specific target loci for whole-genome sequencing data typing, by using the SeqSphere+ target definer tool (Ridom, Munster, Germany) with default options (*19*). As reference, we used the genome of strain NCTC 13129 from the National Center for Biotechnology Information (NCBI) (accession no. BX248353.1/NC_002935.2). We used all 14 complete *C. diphtheriae* genomes available from NCBI as query sequences (accession nos. NC_016782.1, NC_016799.1, NC_016800.1, NC_016801.1, NC_016785.1, NC_016786.1, NC_016802.1, NC_016787.1, NC_016788.1, NC_016783.1, NC_016789.1, NC_016790.1, NZ_LN831026.1, NZ_CP018331). The resulting cg-MLST scheme consisted of 1,553 target loci. An accessory target scheme with 601 more loci was defined during the same process. The targets of the latter were genes not found in each query genome, or they were genes found multiple times in query genomes, overlapping in the reference, or showing an incorrect number of stop codons in >80% of the query genomes.

We performed next-generation–based MLST and cg-MLST with de novo assembled contigs after read-trimming and assembly by using Velvet in SeqSphere+ (Ridom, Munster, Germany) (*20*) with default settings. We performed in silico MLST by using the previously described 7 target loci and cg-MLST by using the generated cg-MLST or extended cg-MLST scheme of 1,553 or 2,154 target loci. After typing and assigning allele numbers, we calculated distances for tree building; during pairwise comparisons of allele profiles, we ignored missing values. Subsequently, minimum spanning trees were generated. We defined a cluster as a group of closely related cg-MLST–analyzed isolates differing by <5 alleles and subclusters with the same similarity threshold but after extended cg-MLST.

For SNP-based phylogeny, NGS reads were adapter-clipped and trimmed for quality and trimmed from short reads <50 bp with trimmomatic (*21*). By mapping against the *C. diphtheriae* reference genome, we performed SNP calling and filtering with the run_snp_pipeline script of the PHEnix pipeline by Public Health England (https://github.com/phe-bioinformatics/PHEnix). This process included bwa-mem mapping (*22*) with default settings and variant calling and filtering (frequency >0.7, mapping quality score >30, read depth >8) by GATK2 Unified Genotyper (*23*). We generated variant call files containing SNP positions passing filters and all positions not passing filters. SNPs were concatenated to FASTA-format alignments with the vcf2fasta-script from the PHEnix pipeline, converting bases at filter-failed positions to letter N, indicating an ambiguous base call, and allowing <90% missing data per sample and <20% missing data at each specific site within the sample set. We generated maximum-likelihood trees from SNP alignments by using RaxML (*24*), including 100 bootstrap replicates, and uploaded sequencing data to the NCBI sequence read archive (https://www.ncbi.nlm.nih.gov/sra; BioProject ID PRJNA416260).

## Results

### Samples and Epidemiologic Data

Our study started with the observation of an increasing number of *C. diphtheriae* isolates submitted to the German Consiliary Laboratory on Diphtheria by diagnostic microbial laboratories or local health authorities from different areas of northern Germany from mid-2016 through early 2017. The geographic regions from which increased *C. diphtheriae* isolates were submitted included Berlin; Hamburg and its surrounding cities Bremen, Bremerhaven, Kiel, and Schwerin; and other cities in northern Germany (Hanover, Dortmund, Bochum, Essen, Leverkusen, and Trier). Among many patients for whom epidemiologic data were available, the most common socioeconomic factors were homelessness, drug or alcohol abuse, or both. Initial routine diagnostic testing, including species identification of *C. diphtheriae* by MALDI-TOF and Coryne API, toxin gene presence testing by quantitative PCR, and random typing by MLST, identified 19 nontoxigenic cases caused by ST8, which led to suspicion of a potential outbreak. We selected those 19 ST8 isolates for NGS analysis, together with samples submitted from the same geographic regions, either from the same period and not yet analyzed by MLST or from an earlier period (2012–2015) and typed as ST8 or not yet typed. A total of 76 nontoxigenic *C. diphtheriae* isolates, submitted from April 2012 through July 2017 from the delineated geographic regions were subjected to the NGS-based in-depth outbreak investigation (Technical Appendix).

The 76 analyzed isolates belonged mainly to biotype gravis (n = 45), followed by biotypes mitis (n = 30) and belfanti (n = 1). Most (63 [83%]) patients were male. For 25 patients, the common socioeconomic characteristics previously identified as risk factors (drug abuse, alcohol abuse, homelessness, or any combination) were confirmed (*6**,**8*). However, because of the retrospective design of the study, for many patients, epidemiologic information was not available or was incomplete. For 19 patients, travel to other countries or origin from other countries was reported. For 7 patients, mixed infections involving several pathogens were reported. Median patient age was 45 years; 38% were 40–60; 37%, 20–40; 13%, >60; and 5%, <20 years of age. Most (61 [80%]) strains were isolated from wound infections (e.g., 7 ulcers, 3 abscesses, 2 phlegmons, 1 deep wound after an amputation, 1 insect bite, 1 scratch, and 1 burn). Two wound infections, located on a patient’s ear and hand, were associated with previous human bites. Most wound infections were reportedly on the lower (n = 26) or upper (n = 15) limbs. For the other patients, no wound specifics were reported. For 9 patients, severe invasive complications had developed, including 1 case of endocarditis, 2 cases of bacteremia, and 6 cases of sepsis. For the remaining patients, the type of infection was either not specified (n = 2) or the report listed diagnoses of single cases of peritonsillar abscess, olecranon bursitis, pharyngitis, tonsillitis, or investigation of carrier status. 

### Whole-Genome Sequencing Results

NGS runs with 2 × 250-bp paired-end reads resulted in 74.5%–79.5% of Q30 bases. An average coverage of 29.3–179.2-fold per sample was obtained; 75 of 76 samples reached coverage of >30, and 73 samples reached coverage of >50.

**Figure 1:**
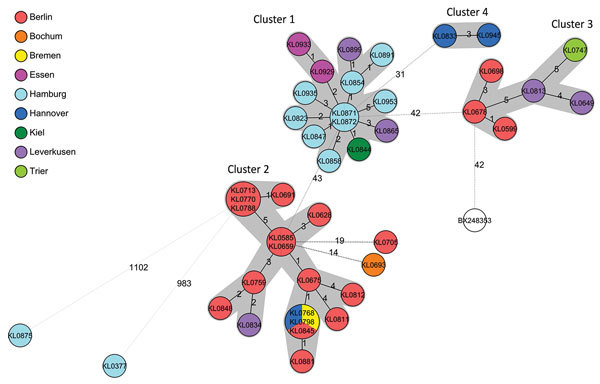
Minimum-spanning tree of core genome multilocus sequence typing with 1,553 targets of the nontoxigenic *Corynebacterium diphtheriae* sequence type 8 (ST8) isolate branch for isolates submitted from northern Germany, January 2015–June 2017. The branch of 41 ST8 typed isolates is shown, together with the 2 nearest isolates from other STs KL0875 (ST441) and KL0377 (ST123) and the reference genome used (GenBank accession no. BX248353). Allelic distances between isolates are indicated, clusters with allele difference <5 are shaded in gray, and the reference genome is shown in white.

NGS reads were assembled de novo, and contigs of yet untyped isolates were analyzed by MLST with the 7-gene scheme (*10*). As expected from the sample selection, among the 20 STs identified, the largest group was formed by ST8 (n = 41 [54%] isolates), consistent with recent observations of ST8 being 1 of the 2 most abundant *C. diphtheriae* STs in PubMLST and thus probably in central Europe (*3*). The next most commonly identified STs were ST130 (10 isolates) and ST439 (5 isolates). All other STs were associated with only 1–5 isolates each (Technical Appendix). cg-MLST with an in-house generated scheme, consisting of 1,553 *C. diphtheriae*–specific target loci, and visualization in a minimum spanning tree revealed that the different STs clustered in ST-specific branches (Technical Appendix Figure). All 41 ST8 isolates were bundled in a distinct branch, separated from all other STs, different from all other isolates by >983 alleles. The ST8 branch was inspected in more detail and showed 4 clearly distinguishable clusters, which came from 2–17 isolates (Figure 1). The maximum difference between the isolates within each cluster ranged from 3 to 5 alleles. Of note, all 9 ST8 isolates from Hamburg belonged to 1 cluster of 14 isolates, together with isolates from Kiel, Essen, and Leverkusen, but none from Berlin. The other 2 bigger clusters with 17 (cluster 2) and 6 (cluster 3) isolates included isolates from Berlin and various other cities (Leverkusen, Bremen, and Hanover in cluster 2 and Leverkusen and Trier in cluster 3) but none from Hamburg. Cluster 4 consisted only of 2 isolates from Hanover, showing a difference of 3 alleles. Clusters 1, 2, and 4 consisted solely of biotype gravis, whereas cluster 3 consisted mainly of isolates of biotype mitis except for 1 isolate that was biotype gravis.

### Enhanced Resolution Analysis by Accessory Targets and SNPs

To enhance resolution, we subsequently analyzed the branch of ST8 isolates showing the 4 described clusters by conducting a second, extended, cg-MLST analysis and whole-genome SNP phylogeny. Therefore, the cg-MLST scheme was enlarged by adding 601 accessory targets to the 1,553 cg-MLST targets. To confirm and further refine results, we performed SNP-based maximum-likelihood phylogeny and generated the phylogenic tree from concatenated SNP sequences after reference-based mapping and variant calling.

**Figure 2:**
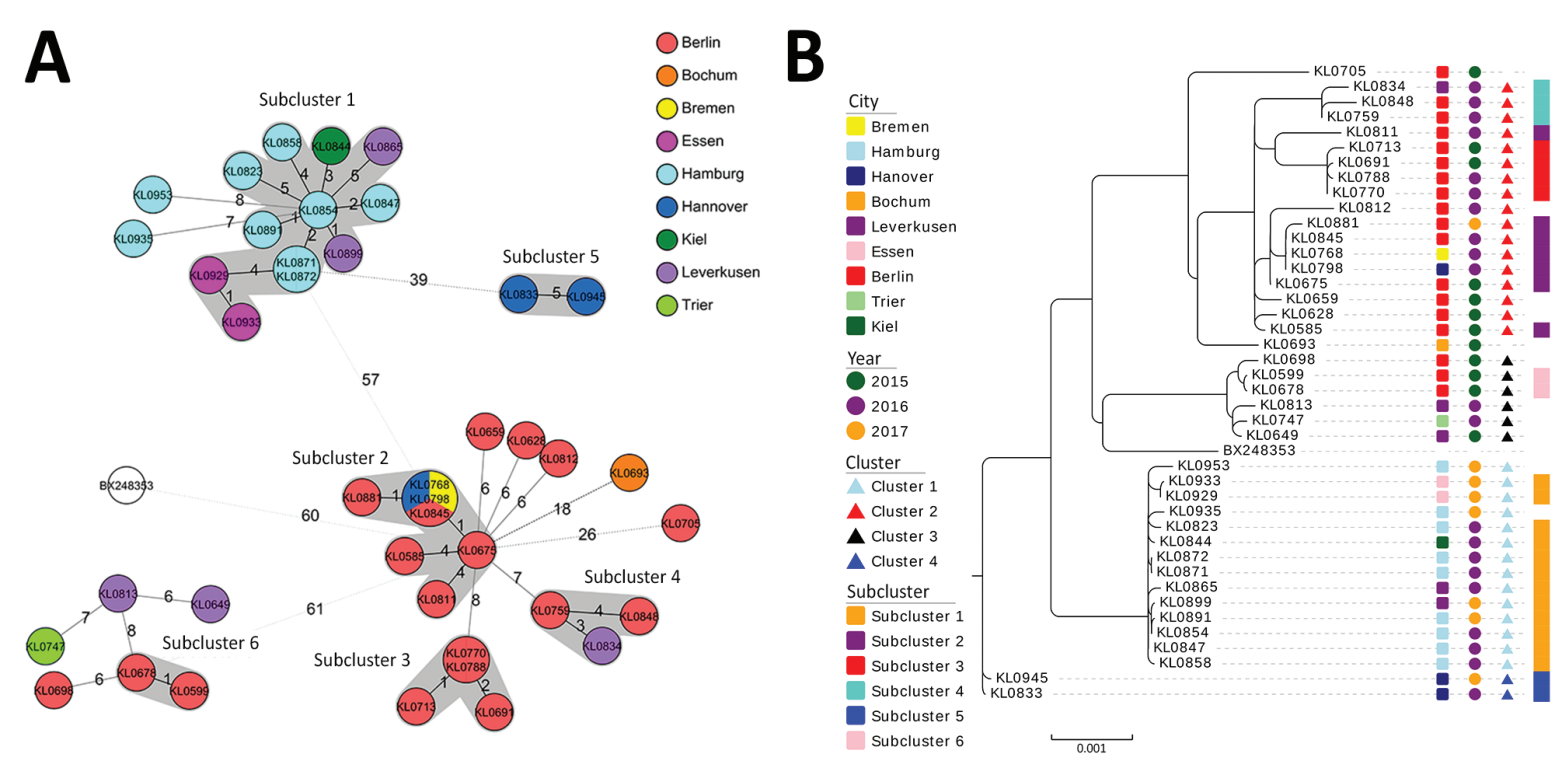
Enhancement of phylogenetic analysis depth of the 41 nontoxigenic sequence type 8 *Corynebacterium diphtheriae* isolates submitted from northern Germany, January 2015–June 2017, and reference genome. A) Phylogenetic minimum-spanning tree of isolates analyzed by extended core-genome multilocus sequence typing (cg-MLST), based on 2,154 target loci. Allele distances between isolates are indicated. Gray indicates subclusters with allele difference <5; white indicates the reference genome (BX248353). B) Phylogenetic maximum-likelihood tree based on genome-wide single-nucleotide polymorphism profiles. From left to right, the color-coded columns indicate isolate affiliations to submitting city, isolate submission year, cg-MLST cluster based on allelic difference of <5 alleles out of 1,553 cg-MLST target loci, and extended cg-MLST-subclusters based on allelic difference of <5 alleles out of 1,553 plus 601 extended cg-MLST target loci. Relative distances are based on 30 000 genomic positions. Scale bar indicates the portion of relative distances based on 14,920 distinct aligned positions.

The general phylogenetic structure of the ST8 branch, with its 4 main clusters, comprising isolates from patients involved in the outbreak, could be confirmed by including more genomic positions in extended cg-MLST (Figure 2, panel A). In addition, the SNP-based phylogenetic tree organizes the isolates in cluster-confirming branches (Figure 2, panel B). The distances within the clusters were generally enhanced by including more target regions. Among the isolates of all 4 clusters, the maximum distance was 124 SNPs. In cluster 1, locally restricted to mainly Hamburg, and in cluster 4 with the 2 isolates from Hanover, the observed similarity of the isolates was still very high. In cluster 1, only the 2 most recent isolates from May and June 2017 drifted from the others with an increased maximum allelic difference of 8, corresponding to 13 SNPs. Considering the cluster threshold of 5 alleles previously defined in cg-MLST, the other 12 isolates still fell within a common cluster named subcluster 1. The maximum difference in cluster 4 was enhanced from 3 to 5 alleles. Distances between isolates of clusters 2 and 3 with a geographic focus in Berlin also increased to 8 alleles, corresponding to 32 SNPs within cluster 2 and 21 SNPs in cluster 3. This higher heterogeneity led to a partial decomposition of clusters 2 and 3. Cluster 2, especially, broke into 3 smaller subclusters (Figure 2, panel A) with 7, 4, and 3 isolates in subclusters 2, 3, and 4, differing in 19, 3, and 10 SNPs, respectively. For isolates KL0811 and KL0698 only, assignment to subcluster 2 could not be confirmed by SNP analysis, indicating, as expected, that the depth of SNP phylogeny was higher than that of extended cg-MLST.

## Discussion

The phylogenetic analysis of the ST8 branch (n = 41 isolates) revealed 4 main outbreak clusters. Generally, the clusters were concentrated around geographic areas, especially Hamburg (cluster 1) and Berlin (clusters 2 and 3). Moreover, there were no clusters in which isolates from Hamburg and Berlin were found together. Three isolates from cluster 2 were genomically identical, although they were submitted from Bremen (KL0768), Hanover (KL0798), and Berlin (KL0845) during March–September 2016. Of note, transmission from 1 city to another occurred and is not surprising because of the considerable travel within the population group.

Generally, clusters 2 and 3, including the isolates from Berlin, seemed to be more heterogeneous and fragmented in smaller subclusters when resolution of analysis was enhanced by including more genes in the cg-MLST scheme or by SNP analysis. In contrast, within the cluster comprising the Hamburg isolates, very high genetic similarity was still noted. It is highly likely that within the 4 main clusters, and especially their resulting subclusters, several direct transmission events have taken place. For example, epidemiologic data proved a direct transmission event for isolates KL0929 and KL0933 in cluster 1, which differed by only 1 allele/1 SNP. They were submitted within 1 week in April 2017 and were isolated from 2 wound infections (from a homeless man and woman from Essen with a proven epidemiologic link). Their high similarity to the remaining isolates from cluster 1, originating mainly from Hamburg, also suggests a tight transmission chain between these isolates. However, for the transmission between Essen and Hamburg, we found no epidemiologic link. Unfortunately, for many patients not enough information was available for us to draw a clear epidemiologic link or to identify further contact persons as part of the transmission chain, even for those with genetically identical isolates. For example, in 2 pairs of isolates, 1 from Hamburg (KL0871, KL0872; 0 SNP distance) and 1 from Berlin (KL0770, KL0788; 1 SNP distance), each pair came from a human bite–associated wound infection of a homeless man and a genetically virtually identical isolate from a limb-located wound infection of another patient. However, no epidemiologic link between the patients was available other than their isolates having been submitted from the same city. It is conceivable that these patients may not have infected their respective counterpart directly but rather that more undetected cases, possibly also of carrier status, may have contributed.

Even if epidemiologic data enabling links to cases are missing, the specific environment of the group of homeless and drug-using patients not only enhances the individual risk of acquiring infection but may also contribute to infection chains (e.g., by the reuse or shared use of needles and lack of hygiene measures) (*25*). For example, several patients had abscesses at a needlestick site. Several isolates of cluster 2 (KL0759, KL0788, KL0812, and KL0845) originated from patients who reportedly lived together in an abandoned house in Berlin, suggesting a common source or mode of transmission.

Pathogenic features such as invasiveness could generally not be connected to a specific outbreak clone. Unfortunately, information about the portal of entry was not submitted for all invasive strains. Wound infections for 4 of 9 patients and pneumonia for 1 patient were reported in addition to the underlying systemic disease. Both isolates from cluster 4 that were genetically similar (isolates KL0833 and KL0945) were responsible for endocarditis and sepsis. The other invasive isolates (KL0507, KL0675, KL0693, KL0768, KL0881, KL0954, KL0964) originated from persons in various cities and at various dates or belonged to different STs; thus, their invasiveness probably depended on host factors.

The more heterogeneous Berlin-focused clusters 2 and 3 were submitted from 2015 through January 2017. In contrast, isolates of homogeneous clusters 1 and 4 were submitted from June 2016 through June 2017. Expanded cg-MLST and SNP-based phylogeny showed a less close relationship for clusters 2 and 3, with isolates submitted from an earlier and longer time frame, than within clusters 1 and 4, with isolates submitted from a later and shorter time frame. For several isolate connections within the clusters, a chronological order could be assumed from the SNP-based phylogenetic tree (e.g., between isolates KL0675, KL0768/KL0798/KL0845, and KL0881 in cluster 2) (Figure 2, panel B). To visualize these ordered connections, inclusion of earlier isolates in the dataset proved useful. Even in the very homogeneous cluster 1, the most recent isolates KL0935 and KL0953 veered away in the extended cg-MLST analysis (Figure 2, panel A) and showed an enhanced branch length in the maximum-likelihood tree (Figure 2, panel B). These observations show hints for evolution of the outbreak strains. However, it seems that evolution proceeded rather slowly because allele and SNP profiles showed high similarities in related isolates over longer periods of several months to almost a year (e.g., clusters 1 and 4). However, not much is known about distances characterizing clonal complexes or parentage within the genus *Corynebacterium*. As an example of a non–outbreak-related ST8 isolate, we included the used reference genome in cg-MLST and SNP phylogeny, which is a UK-derived representative of the former Soviet Union outbreak in the 1990s (*26*). This isolate showed a similar allelic difference to the 4 clusters as did the clusters between each other and was arranged at a superordinate branch of cluster 3 in the SNP dendogram. We concluded that the 4 geographically concentrated clusters have probably been evolving separately for years. To classify isolates more clearly into common or separated ancestorship in the future, allelic or SNP difference threshold definitions for clonal complexes would be of great advantage. To gain the underlying knowledge, more sequencing studies of carefully selected isolate sets will be needed.

In conclusion, by whole-genome sequencing analysis, we identified several outbreak clusters of nontoxigenic *C. diphtheriae* overlapping with specific geographic areas in metropolitan areas of northern Germany. Direct transmission between patients probably occurred within these local clusters. In contrast, the different clusters seem to have been separated from each other for years. Nonetheless, outbreak strains are persisting and show an ongoing, although slow, evolution. Our stepwise, high-resolution approach for NGS-based outbreak analysis of nontoxigenic *C. diphtheriae* showed that MLST analysis can still serve as a good first-level molecular analysis and standard classification (e.g., to confine possible outbreak candidates). At the next level, whole-genome sequencing brings deeper and more comprehensive insights during the investigation of *C. diphtheriae* outbreaks causing different clinical symptoms and distributed over wider geographic areas and longer periods. We also show that nontoxigenic *C. diphtheriae* can become a public health threat in industrialized countries because strains can persist, evolve within risk groups, and lead to outbreaks, which are difficult to register early enough to stop transmission. We suspect that this threat is not specific for Germany only but that it is a potential problem for specific risk groups in metropolitan areas in general. Therefore, clinicians and public health authorities should bear in mind the potential for nontoxigenic *C. diphtheriae* to cause disease (*7**,**8**,**27**,**28*) and should recognize infection and transmission events early.

Technical Appendix Nontoxigenic *Corynebacterium diphtheriae* isolates selected for next-generation sequencing; minimum spanning tree of core-genome multilocus sequence typing with 1,553 targets of all 76 nontoxigenic *C. diphtheriae* isolates submitted from northern Germany, 2016–2017.
