# Supplementary material for: Geographically Diverse Clusters of Nontoxigenic Corynebacterium diphtheriae Infection, Germany, 2016–2017
Source: Emerg Infect Dis. 2018 Jul;24(7):1239–45. doi: 10.3201/eid2407.172026 (PMC6038752; doi:10.3201/eid2407.172026)
Supplement: Technical Appendix — Nontoxigenic Corynebacterium diphtheriae isolates selected for next-generation sequencing; minimum spanning tree of core-genome multilocus sequence typing with 1,553 targets of all 76 nontoxigenic C. diphtheriae isolates submitted from northern Germany, 2016–2017. [file 17-2026-Techapp-s1.pdf]

Article DOI: https://doi.org/10.3201/eid2407.172026

Geographically Diverse Clusters of Nontoxigenic *Corynebacterium diphtheriae* Infection, Germany, 2016–2017

Technical Appendix

Technical Appendix Table. Non-toxigenic *C. diphtheriae* isolates from Northern Germany accumulations, selected for NGS analysis*

Sample ID	Receipt Date	Isolation Source	Disease	City	Age	Sex	Biovar	Sequence type	Risk factors/ Travel history
KL0330	05.04.2012	Leg	Wound infection	Hamburg	62	Male	Mitis	130	Homeless
KL0355	04.07.2012	Foot	Wound infection	Hamburg	29	Male	Mitis	494	NA
KL0360	18.07.2012	Skin	Wound infection	Hamburg	73	Male	Mitis	297	Africa
KL0371	20.09.2012	Back	Wound infection	Hamburg	38	Male	Mitis	130	NA
KL0372	02.10.2012	Head	Wound infection	Hamburg	47	Male	Mitis	130	Mixed infection (*S. Aureus*, *Enterobacter sp.*, *Pantoea sp.*)
KL0377	30.10.2012	Skin	Wound infection	Hamburg	23	Male	Gravis	123	NA
KL0434	25.07.2013	Skin	Wound infection, insect bite	Bremerhaven	4	Male	Mitis	259	Travel activity, destination not reported
KL0438	13.08.2013	Skin	Wound infection	Hamburg	48	Female	Mitis	130	NA
KL0461	23.10.2013	Hand	Wound infection	Hamburg	18	Male	Mitis	495	NA
KL0476	10.12.2013	Foot	Wound infection	Hamburg	68	Male	Gravis	295	Travel activity, destination not reported
KL0479	12.12.2013	Skin	Wound infection	Bremerhaven	53	Male	Belfanti	501	Hepatitis C
KL0507	09.04.2014	Blood	Sepsis	Hamburg	46	Male	Mitis	130	NA
KL0522	14.05.2014	Foot, leg	Wound infection	Hamburg	29	Male	Mitis	439	Drug abuse, mixed infection (*S. Pyogenes*, *S. Aureus*)
KL0557	09.09.2014	Leg	Wound infection	Hamburg	35	Female	Gravis	497	Sri Lanka, mixed infection (*S. Pyogenes*)
KL0585	23.01.2015	Leg	Wound infection	Berlin	35	Male	Gravis	8	NA
KL0599	30.03.2015	Leg	Wound infection, abscess	Berlin	45	Male	Mitis	8	Homeless
KL0628	12.06.2015	Tonsils	Tonsillitis	Berlin	29	Male	Mitis	8	NA
KL0637	30.06.2015	NA	NA	Hamburg	66	Male	Mitis	498	NA
KL0649	10.07.2015	Leg	Wound infection	Leverkusen	35	Female	Gravis	8	Origin: Belgium
KL0659	05.08.2015	Foot	Wound infection	Berlin	63	Male	Gravis	8	Ketoacidotic coma
KL0660	06.08.2015	Foot	Wound infection	Hamburg	71	Male	Mitis	130	NA
KL0675	02.09.2015	Arm	Wound infection, sepsis, phlegmon	Berlin	NA	Male	Gravis	8	Homeless
KL0676	04.09.2015	Skin	Wound infection	Hamburg	51	Male	Mitis	439	NA
KL0678	08.09.2015	Foot	Wound infection	Berlin	58	Male	Mitis	8	Homeless, Alcohol abuse
KL0691	09.10.2015	Finger	Wound infection	Berlin	42	Male	Gravis	8	Homeless
KL0693	13.10.2015	Blood	Sepsis, pneumonia	Bochum	53	Male	Gravis	8	Origin: Poland
KL0698	26.10.2015	Knee	Wound infection	Berlin	62	Male	Mitis	8	NA
KL0705	23.11.2015	Skin	Wound infection, ulcer	Berlin	82	Male	Gravis	8	NA
KL0713	17.12.2015	Hand	Wound infection	Berlin	38	Male	Gravis	8	NA

Sample ID	Receipt Date	Isolation Source	Disease	City	Age	Sex	Biovar	Sequence type	Risk factors/ Travel history
KL0747	20.01.2016	Elbow	Wound infection	Trier	45	Male	Mitis	8	Homeless, Drug abuse
KL0759	08.02.2016	Elbow	Olecranon bursitis	Berlin	31	Male	Gravis	8	Homeless
KL0762	02.03.2016	Throat	Pharyngitis, tonsillitis	Hamburg	33	Male	Mitis	446	NA
KL0768	10.03.2016	Blood	Sepsis, endocarditis	Bremen	56	Male	Gravis	8	Homeless
KL0770	16.03.2016	Leg	Wound infection, ulcer	Berlin	51	Male	Gravis	8	NA
KL0788	08.04.2016	Hand	Wound infection, phlegmon, post human bite	Berlin	37	Male	Gravis	8	Homeless, Origin: Poland
KL0798	10.05.2016	Skin	Wound infection, burn	Hanover	39	Male	Gravis	8	Homeless
KL0811	25.05.2016	Leg	Wound infection	Berlin	40	Male	Gravis	8	Homeless, alcohol abuse, origin:Poland
KL0812	27.05.2016	Arm	Wound infection, abscess	Berlin	20	Male	Gravis	8	NA
KL0813	30.05.2016	NA	Carrier	Leverkusen	36	Female	Mitis	8	NA
KL0816	02.06.2016	Leg	Wound infection	Bremerhaven	54	Male	Mitis	499	NA
KL0823	28.06.2016	Wound	Wound infection, scratch	Hamburg	30	Male	Gravis	8	Homeless, Alcohol abuse
KL0833	08.08.2016	Blood	Sepsis (initial finding: wound infection)	Hanover	57	Male	Gravis	8	Homeless
KL0834	18.08.2016	Foot	Wound infection	Leverkusen	56	Female	Gravis	8	Drug abuse
KL0844	28.09.2016	Skin	Wound infection	Kiel	48	Male	Gravis	8	NA
KL0845	29.09.2016	Sub facial	Wound infection	Berlin	31	Female	Gravis	8	NA
KL0847	30.09.2016	Thumb	Wound infection	Hamburg	43	Male	Gravis	8	NA
KL0848	07.10.2016	Punctate	Abcess peritonsillar	Berlin	45	Male	Gravis	8	Origin:Russia
KL0854	26.10.2016	Hand	Wound infection, tissue	Hamburg	36	Male	Gravis	8	Origin:Nepal
KL0858	08.11.2016	Hand	Wound infection	Hamburg	40	Male	Gravis	8	NA
KL0865	12.12.2016	Hand	Wound infection	Leverkusen	38	Female	Gravis	8	NA
KL0871	28.12.2016	Hand	Wound infection	Hamburg	42	Female	Gravis	8	Mixed infection (*S. Pyogenes*), Origin: Hungary
KL0872	30.12.2016	Ear	Wound infection, post human bite	Hamburg	57	Male	Gravis	8	Homeless, Alcohol abuse
KL0875	05.01.2017	Foot	Wound infection	Hamburg	26	Male	Gravis	441	Sri Lanka, doctor
KL0881	13.01.2017	Blood	Sepsis (initial finding: wound infection)	Berlin	51	Male	Gravis	8	Homeless, Alcohol abuse, origin: Croatia
KL0888	16.01.2017	Ankle	Wound infection, ulcer	Hamburg	75	Male	Mitis	389	Mixed infection (*S. Pyogenes*, *E. Coli*)
KL0891	18.01.2017	Leg	Wound infection	Hamburg	19	Male	Gravis	8	Homeless, drug abuse
KL0895	23.01.2017	Foot	Wound infection	Hamburg	63	Male	Gravis	295	Sri Lanka, mixed infection (MRSA)
KL0899	30.01.2017	Skin	Wound infection	Leverkusen	31	Male	Gravis	8	NA
KL0920	14.03.2017	Hip	Wound infection	Schwerin	30	Male	Mitis	294	Origin: Eritrea
KL0929	11.04.2017	Knee	Wound infection	Essen	44	Male	Gravis	8	Homeless
KL0932	13.04.2017	Finger	Wound infection, ulcer	Schwerin	53	Female	Gravis	130	Homeless
KL0933	19.04.2017	Shoulder	Wound infection	Essen	56	Female	Gravis	8	Homeless
KL0935	11.05.2017	Knee	Wound infection, arthritis	Hamburg	37	Female	Gravis	8	Homeless, mixed infection (MRSA)
KL0936	15.05.2017	Penis	Wound infection, ulcer	Berlin	67	Male	Gravis	441	India

Sample ID	Receipt Date	Isolation Source	Disease	City	Age	Sex	Biovar	Sequence type	Risk factors/ Travel history
KL0937	15.05.2017	Thigh	Wound infection, deep wound after amputation	Berlin	46	Male	Mitis	130	NA
KL0939	18.05.2017	Leg	Wound infection	Berlin	40	Male	Mitis	130	NA
KL0940	19.05.2017	Skin	Wound infection, ulcer	Hamburg	65	Male	Mitis	507	NA
KL0943	29.05.2017	NA	NA	Hamburg	49	Male	Mitis	130	NA
KL0945	30.05.2017	Blood	Endocarditis, aortic valve abcess	Hanover	39	Male	Gravis	8	Origin: Poland
KL0947	31.05.2017	Arm	Wound infection, abscess	Hamburg	36	Male	Mitis	439	Origin: Turkey
KL0952	19.06.2017	Skin	Wound infection, ulcer	Berlin	40	Male	Gravis	508	NA
KL0953	19.06.2017	Leg	Wound infection	Hamburg	52	Female	Gravis	8	Homeless
KL0954	23.06.2017	Blood	Bacteraemia	Berlin	76	Male	Mitis	439	Homeless, drug abuse
KL0961	04.07.2017	Foot	Wound infection	Leverkusen	54	Male	Gravis	509	Origin: Sri Lanka
KL0964	06.07.2017	Blood	Bacteraemia, (initial finding: wound infection)	Berlin	48	Male	Mitis	439	Homeless
KL0965	12.07.2017	Skin	Wound infection	Dortmund	52	Female	Gravis	295	Sri Lanka

*Epidemiologic data included as available (NA = no information available).

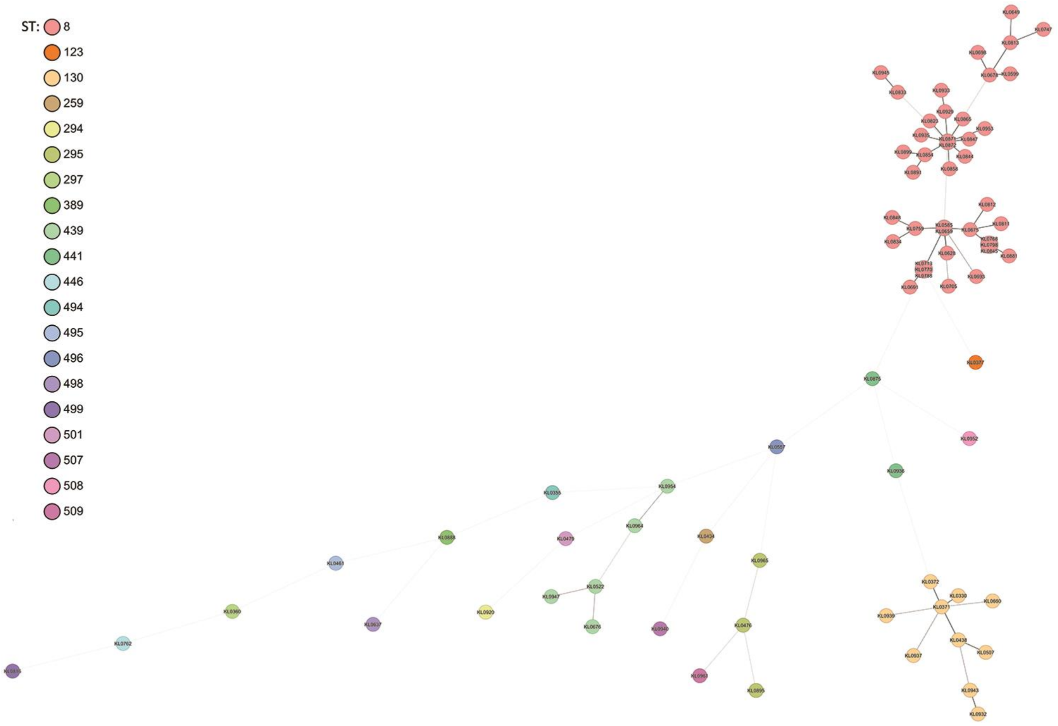

Technical Appenedix Figure. Minimum spanning tree of core-genome multilocus sequence typing with 1,553 targets of all 76 nontoxigenic *Corynebacterium diphtheriae* isolates submitted from northern Germany, April 2012–July 2017, and analyzed by next-generation sequencing and typed by 1,553 core-genome multilocus sequence targets. Samples cluster in sequence type–specific branches and are colored by sequence type affiliation.
